# Negative coping style mediates the relationship between negative mental and suicide risk among migrant workers in China

**DOI:** 10.1038/s41598-021-03888-3

**Published:** 2022-01-10

**Authors:** Han Xiao, Xiaoyi Li, Zhijian Zhou, Huiming Liu, Chiyi Hu, Tiebang Liu, Dafang Chen, Liqing You

**Affiliations:** 1 Department of Public Health, Peking University Health Science Centre, Beijing, 100091 China; 2 Shenzhen Kangning Psychiatric Hospital, Shenzhen, 518020 Guangdong China

**Keywords:** Psychology, Human behaviour

## Abstract

Suicide is increasingly recognized as a major public health concern among migrant workers in China. Despite negative mental and negative coping styles being core themes found in suicide notes, there is scarce research addressing the theoretical framework of underlying mechanisms between these variables. The study was designed to examine the relationships of negative mental, negative coping styles, and suicide risk among migrant workers. It hypothesized that negative mental would exert a positive effect on suicide risk via increased negative coping. Using a cross-sectional design, the study was conducted using a sample of 3095 migrant workers from Shenzhen, China. Self-made Suicide Risk Scale (SRS), Short-form of the ULCA Loneliness Scale (USL-6), Patient Health Questionnaire-9 (PHQ-9), Generalized Anxiety Scale (GAD-7), Simplified Coping Style Questionnaire (SCSQ) were used to collect data. Structural equation modeling (SEM) was performed to quantitatively explore the path effects between negative mental, negative coping styles and suicide risk. Results showed that negative coping style had a positive association with suicide risk (β = 0.029, P < 0.001). Negative mental had both direct and indirect positive effects on suicide risk through negative coping styles (β = 0.109, β = 0.013, P < 0.001). Therefore, to prevent suicidal behaviors among migrant workers, targeted interventions focusing on improving their mental health and coping strategies are needed.

## Introduction

Since the Chinese government has implemented the system of residential registration from in 1958, each citizen was categorized as having an “urban” or “rural” belonging at birth^1^. The policy was intended to restrict the inflow of rural populations into cities. However, China has still witnessed a significant demographic transition during the past three decades, characterized as rural-to-urban workers migrating to cities due to job opportunities and favorable income. Migrant workers refer to farmers who leave their original residence in rural areas and work in cities for 6 months or more^2^. According to population data, there were 286.54 million migrant workers in China in 2017, making up approximately one-fifth of the entire Chinese population^3^.

On the one hand, numerous migrant workers in China provide cities with, abundant cheap labor and accelerate the economic growth. On the other hand, because of the Chinese system of household registration (*hukou)*, the majority of these migrant workers without urban household registration are classified as temporary residents in cities, irrespective of their length of stay. Unlike permanent city residents, migrant workers have to move back and forth between their hometowns where they are considered to be permanent residents and the cities where they temporarily work and dwell^4^. Moreover, migrant workers with *hukou* restrictions do not have the same access to benefits such as employment, labor security, health insurance, social welfare, housing and educational opportunities that are available for registered urban citizens^5^. In brief, they experience various forms of policy exclusion, economic exclusion and even psychological exclusion such as social stigma and discrimination^6^. Therefore, migrant workers under the aforementioned pressures in a cross-cultural social environment were often considered to be more vulnerable to mental illness and suicidal behaviors^7^.

In the year of 2010, 18 migrant workers from Foxconn Technology Group in Shenzhen attempted suicide, arousing public attention to the poor mental health status of migrant workers in China. Most research has demonstrated that migrant workers were more susceptible to a variety of negative mental such as depression, loneliness and anxiety. For example, data from studies using Mini International Neuropsychiatric Interview demonstrated that 5.08% of the migrant workers in Shenzhen experienced major depressive disorders during their lifetime^4^. Zhong et al. (2016) found that 18.3% of migrant workers suffered from loneliness due to social isolation^8^. Similarly, another study in Shenzhen reported the prevalence of anxiety was 19.26% among migrant workers^9^. Evidence from China also showed that migrant workers were more likely to commit suicides compared to nonmigratory rural residents^10^.

Previous research has reported that negative mental such as depression, loneliness and anxiety are significant predictors of suicidal behaviors in adolescents^11–13^. Correspondingly, negative mental was also found closely associated with suicidal behaviors among migrant workers. For instance, a retrospective study among migrant workers in Shenzhen, China asserted that migrant workers with mental disorders were at higher risk for developing suicidal behaviors^14^. Another cross-sectional study based on 1845 migrant workers in China indicated that negative mental such as depression and anxiety played a direct role on suicidal behavior^15^. Based on the stress-diathesis model of suicidal behaviors (SDSB), negative mental often served as personal diathesis which directly contributed to suicidal behaviors^16^. Specifically, migrant workers who had experienced higher levels of stress would have higher levels of negative mental, it is reasonable to propose that the higher level of negative mental would eventually increase the risk of committing suicide among migrant workers.

Negative coping styles are known as moderating factors between life stress and suicidal behaviors based on the strain theory of suicide^17^. Negative coping may also affect suicide risk among migrant workers in a Chinese context. For instance, a cross-sectional study among Chinese rural youth reported that negative coping style was significantly associated with suicidal ideation^17^. Similarly, Sun et al., found that negative coping can increase the risk of suicide among Chinese rural youth^18^. Previous studies have shown that rural-to-urban migrants suffer from lower mental health status than urban counterparts^19^. However, migrants lacking in coping skills might tend to solve the psychological suffering passively, which leads to a higher risk of committing suicide.

Though most previous studies examined negative mental and negative coping styles as risk factors for suicidal behaviors, structural pathways linking these variables have not been examined among migrant workers in China. Negative coping style may serve as a mediator since there is also a significant association between negative mental health and negative coping. For instance, a study using a sample of Latino migrant workers found that stressed or depressed workers are more likely to pursue negative coping behaviors^20^.

The current study was thus designed to address the knowledge gap on the relationships between negative mental, negative coping styles and suicide risk. Furthermore, we sought to inform prevention strategies by better understanding the potential psychological mechanism of suicide. Based on prior theories and research, we proposed a theoretical model. We hypothesized that negative mental, including depression, anxiety and loneliness, would be positively associated with suicide risk (*hypothesis 1*). We predicted that negative coping styles would be positively associated with the suicide risk (*hypothesis 2*). Pursuing the causal chain, we further hypothesized that negative mental would be indirectly associated with suicide risk through negative coping styles (*hypothesis 3*).

## Materials and methods

### Participants

We conducted a cross-sectional study in Shenzhen, China, between September 2018 and June 2018. Migrant workers from 12 enterprises in Shenzhen were invited to participate. Eligible subjects were included in the study if they (a) were aged over 16, (b) hold a rural *hukou* (registered as permanent residence in rural area), (c) have been engaged in the secondary or tertiary industry for at least 6 months in Shenzhen without a Shenzhen permanent *hukou*, (d) were voluntarily in this investigation. Migrant workers were excluded from the study if they were unable to finish the questionnaire (e.g., visual impairments).

Participants were recruited through the multistage clustered random sampling method. First-stage sampling divided the companies of Shenzhen into two strata (outside the Shenzhen Special Economic Zone, inside the Shenzhen Special Economic Zone). The second stage randomly selected 2 companies of each stratum to participate and a total of 8 companies were successfully recruited. In the third stage, 2–4 departments were randomly selected from each included company according to the probability proportionate to size sampling (PPS). All migrant workers of the chosen departments were invited to take part in the survey.

The sample size was calculated according to the formula *N*=$\frac{{\mu }_{\alpha }^{2}\rho (1-\rho )}{{\delta }^{2}}$,where *N* = sample size to be calculated; ${\mu }_{\alpha }$=value of the normal curve associated to the confidence level; $\rho$=expected percentage of the response variable; $\delta$=accepted margin of error^21^. Assuming a prevalence of suicidal behaviors in migrant workers of 11%^22,23^, using $\alpha$ of 0.05, we calculated a required sample size of 3200, with a 20% expansion to allow for invalid samples. A total of 3095 effective samples were used in the analysis with a valid rate of 96.47%. We then compared valid samples and invalid samples (including subjects lost to follow-up and those who failed the questionnaire) with the consistency test. There was no statistically significant difference between the two in gender and age distribution (all *Ps* > 0.05). The study protocol was approved by the Medical Ethics Committee of Shenzhen. All participants provided written informed consent in the survey. The authors assert that all procedures were carried out in accordance with the relevant guidelines and regulations.

### Measures

#### Self-designed socio-demographic questionnaire

Participants provided information on basic socio-demographic characteristics including gender, age, education, marital status (married, unmarried, widowed/divorced), average monthly income in Chinese currency Yuan (CNY), religious beliefs, resident manner (alone, with family, living in community), smoking, excessive drinking and mental health services (one question about MW’s accessibility of mental health services: “Are there any special mental health service agencies in your factory or community to provide you with mental health services?”).

#### Simplified Coping Style Questionnaire (SCSQ)

The SCSQ is a self-report survey that was developed to assess coping styles^24^.This scale contains 20 items using a Likert 4-point scoring method, ranging from 0 (never) to 3 (always).The scale uses two subscales including positive coping styles (12 items; e.g., talking about troubles for help) and negative coping styles (8 items; e.g., trying to forget).Items of each subscale were summed up to generate a total score with a higher total score indicating a higher tendency for corresponding coping styles^24^.In the present study, the *Cronbach’*$s \alpha$ of scale was 0.864.

#### Short-form of the ULCA Loneliness Scale (USL-6)

The USL-6 is a revised version based on the USL-8 after excluding two reversed items as follows: “I am a person who was willing to make friends.” , “I can find someone to accompany me when I want.”^25^. This scale contains 6 items with each item rated on a 4-point scale that measures the degree of loneliness, and the total scores range from 4 to 24, with a higher total score indicating a higher rate of loneliness. The USL-6 has demonstrated excellent validity and reliability in samples of elderly^25^. In the present study, the *Cronbach’*$s \alpha$ of the scale was 0.865.

#### Patient Health Questionnaire (PHQ-9)

The PHQ-9 is a self-report survey that was developed to assess depression^26^. This scale contains 9 items representing the criterion symptoms for DSM-5 major depressive disorder^27^. Participants were asked how much each symptom has bothered them over the past 2 weeks. Using a Likert 4-point scoring method, each item ranges from 0 (not at all) to 3 (nearly every day), and total scores range from 0 to 27 with higher scores representing more severe depression. In the present study, the *Cronbach’*$s \alpha$ of the scale was 0.877.

#### Generalized Anxiety Scale (GAD-7)

The GAD-7 is a self-report survey that was developed to detect generalized anxiety disorder^28^. This scale contains seven items with each item rated on a 4-point scale, and can be scored as a continuous variable from 0 to 21, with higher scores representing more severe anxiety. In the present study, the *Cronbach’*$s \alpha$ of the scale was 0.904.

#### Self-designed Suicide Risk Scale (SRS)

The SRS is designed for this survey to detect each respondent’s suicide risk. In the present study, suicide risk is defined as individual’s risk of suicidal death in the future, which is assessed by the occurrence of suicide-related behaviors, including non-suicidal self-harm behaviors, suicidal ideation and suicide attempts. According to the Ideation-to-action Framework, suicidal ideation and suicide attempts as suicidal behaviors can predict suicide death^29^. Similarly, non-suicidal self-harm behavior was found as a risk factor of suicide death^30^. Therefore, people with suicide-related behaviors are considered high-risk for suicide. In the design of the SRS scale, non-suicidal self-harm behaviors, suicidal ideation, and suicide attempts are all included in the suicide risk measurement.

This scale contains five items with each item rated on a 2-point scale (0 = *yes*, 1 = *no*).The SRS has three subscales: Non-suicidal self-harm behavior(2 items; “whether there has been any non-suicidal self-harming behavior in the past”, “whether there has been any non-suicidal self-harm behavior in the recent or 12 months”); Suicidal ideation(2 items; “Have you ever considered ending your life”, “have you ever made a suicide plan”); Suicidal attempts(“Have you ever committed suicide”). When assessing descriptive analysis and logistic regression models in the present study, each item was coded as dichotomous outcomes such that responses of “no” were coded as 0 and responses with any suicide-related behavior were coded as 1, reflecting having suicide risk. When constructing the structural equation modeling later, three subscales of the SRS were used to assess the latent variable “*suicide risk*” together. In the present study, the *Cronbach’*$s \alpha$ of scale was 0.694.

### Statistical analyses

All analyses were conducted using SPSS version 23.0 and R version 3.6.3^31^, the statistical significance level was set at *p* < 0.05 (two-sided). Socio-demographic factors (e.g., age, gender, education) were examined by suicide risk status (operationalized as 0 or 1 score on SRS) using chi-square (${\chi }^{2}$)test. Descriptive univariate and multivariable logistic regression analyses were used to explore the relationships between negative mental, negative coping styles and suicide risk, adjusting for statistically significant socio-demographic information. Pearson linear correlation coefficients were conducted to examine associations among the study variables.

Finally, generalized structural equation modeling (SEM) tested the theoretical model of the relationship between negative mental, negative coping styles and suicide risk. The outcome variable for the proposed model was “suicide risk”, as measured by 3 subscale scores on the SRS. The key predictor was “negative mental” operationalized as a latent factor with three indicators: depression, anxiety and loneliness. For the SEM, each indicator was coded such that a higher score indicates poorer mental health. Our hypothesized mediator, negative coping styles, was represented by the total scores of the items 13–20 on SCSQ.

Model fit was assessed using the following fit indices: *Root Mean Square Error of Approximation* (*RMSEA*), *Comparative Fit Index* (*CFI*), *Goodness-of-Fit Index* (*GFI*), *Adjusted Goodness-of-Fit Index* (*AGFI*). RMSEA $\le$ 0.05, CFI > 0.90, GFI > 0.90 and AGFI > 0.90 demonstrate a good fit to the model^23^. The relative chi square (CMIN/DF) was also used as a measure of model fit with a smaller value indicating a better fit^32^. The R software with lavaan was used to estimate the structural equation modeling using the weighted least squares mean and variance-adjusted method (WLSMV)^33,34^.

## Results

### Demographic characteristics

A total of 3095 migrant workers completed the survey with 87 workers (2.81%) reported suicide risk. Among them, 23 workers (0.74%) reported suicidal self-harm behaviors, 74 workers (2.39%) reported some suicidal ideation, 11 workers (0.36%) reported suicide attempts.

The socio-demographic characteristics of the subjects with and without suicide risk were summarized in Table 1. Compared to migrant workers without suicide risk, those with suicide risk were more likely to be younger and unmarried, have higher education level, live alone, drink alcohol excessively, have religious belief and do not understand mental health services.

**Table 1:** Comparison of suicide risk among migrant workers with different socio-demographic characteristics [n (%)].

Variables	All respondents	Suicide risk No. (rate, %)	*OR (95%CI)*	*χ*^2^	*P*
**Sex**				0.89	0.345
Men	2032	53 (2.6%)	1.00		
Women	1063	53 (3.2%)	0.81 (0.52–1.26)		
**Age (years)**				6.12	0.047
< 30	757	28 (3.7%)	1.00		
30–45	1642	48 (2.9%)	0.78 (0.49–1.26)		
> 45	696	11 (1.6%)	0.42 (0.21–0.85)		
**Education level**				8.16	0.004
Junior high school and below	1522	35 (2.3%)	1.00		
High school or technical secondary school	1346	37 (2.7%)	1.20 (0.75–1.92)		
College and above	227	15 (6.6%)	3.01 (1.61–5.60)		
**Marital status**				10.52	0.005
Unmarried	1036	43 (4.2%)	1.00		
Married	1959	41 (2.1%)	0.49 (0.32–0.76)		
Divorced or widowed	100	3 (3.0%)	0.71 (0.22–2.35)		
**Resident manner**				14.09	0.001
Live alone	460	25 (5.4%)	1.00		
Live with family	1356	29 (2.1%)	0.38 (0.22–0.66)		
Live in community	1279	33 (2.6%)	0.46 (0.27–0.78)		
**Average monthly income**				1.05	0.305
< 3000	539	17 (3.2%)	1.00		
3000–5000	1995	58 (2.9)	0.92 (0.53–1.59)		
> 5000	561	12 (2.1%)	0.67 (0.32–1.42)		
**Smoke**				1.46	0.226
Yes	966	22 (2.3%)	1.00		
No	2064	65 (3.1%)	1.35 (0.83–2.21)		
**Excessive drinking**				23.31	< 0.001
Yes	1318	59 (4.5%)	1.00		
No	1777	28 (1.6%)	0.34 (0.22–0.54)		
**Mental health service**				11.2	0.004
Yes	673	7 (1.0%)	1.00		
No	1292	38 (2.9%)	2.88 (1.28–6.49)		
Don’t know	1130	42 (3.7%)	3.67 (1.64–8.22)		
**Religious belief**				5.12	0.023
Yes	206	11 (5.3%)	5.30		
No	2889	76 (2.6%)	0.48 (0.25–0.92)		

### Correlation of negative mental, negative coping and suicide risk

Pearson correlation analysis results showed that scores on all mental scale significantly correlated with each other (*P* < 0.05). Specifically, the total score of negative coping style was moderately positively correlated with USL-6, GAD-7, and PHQ-9 (*r* = 0.275, *r* = 0.272, *r* = 0.249, *P* < 0.05). USL-6 was positively correlated with PHQ-9 and GAD-7 (*r* = 0.551, *r* = 0.726, *P* < 0.05). There were also a positive correlation between PHQ-9 and GAD-7 (*r* = 0.569, *P* < 0.05).

### Association among negative mental, negative coping and suicide risk

Associations between suicide risk and depression, anxiety, loneliness and negative coping were summarized in Table 2. The univariate logistic analysis revealed that depression (OR = 1.17), anxiety (OR = 1.19), loneliness (OR = 1.32), and negative coping (OR = 1.12) were significantly positively associated with elevated prevalence of suicide risk. Multivariate logistic model was run after making appropriate adjustments for significant socio-demographic variables. As shown in Table 2, depression (OR = 1.29), anxiety (OR = 1.18), loneliness (OR = 1.15), and negative coping (OR = 1.11) were associated with increased odds of suicide risk.

**Table 2:** Adjusted and unadjusted association of mental scale scores with suicide risk among migrant workers d$(\overline{x }\pm s)$.

Variable	Suicide risk (n = 3008)	No suicide risk (n = 87)	*P*	*OR (95%CI)* ^a^	*P*	*OR (95%CI)* ^b^
Depression	9.74 ± 3.75	15.06 ± 4.18	< 0.001	1.32 (1.254–1.382)	< 0.001	1.29 (1.23–1.36)
Loneliness	3.29 ± 4.43	8.76 ± 5.26	< 0.001	1.17 (1.129–1.201)	< 0.001	1.15 (1.114–1.188)
Anxiety	2.19 ± 3.43	6.09 ± 4.56	< 0.001	1.19 (1.147–1.238)	< 0.001	1.18 (1.128–1.223)
Negative coping	8.05 ± 4.69	11.10 ± 4.69	< 0.001	1.12 (1.079–1.165)	< 0.001	1.11 (1.068–1.160)

USL-6, Short-form of the ULCA Loneliness Scale; PHQ-9, Patient Health Questionnaire; GAD-7, Generalized Anxiety Scale; Negative coping style is the total score of items 13–20 on SCSQ scale. ^a^ univariate logistic analysis; ^b^multiple Logistic analysis adjusted for age, education, marital status, religious belief, mental health service, living status, and alcoholism.

### Test for common method biases

Common method biases were then tested using Harman’s univariate analysis before constructing the path model. All factors with eigenvalues above 1 were identified, accounting for 22.086% of the total variation, which was smaller than the criterion of 40%^35^. This indicated an absence of severe common method biases in the present study.

### SEM of negative mental, negative coping and suicide risk

**Figure 1:**
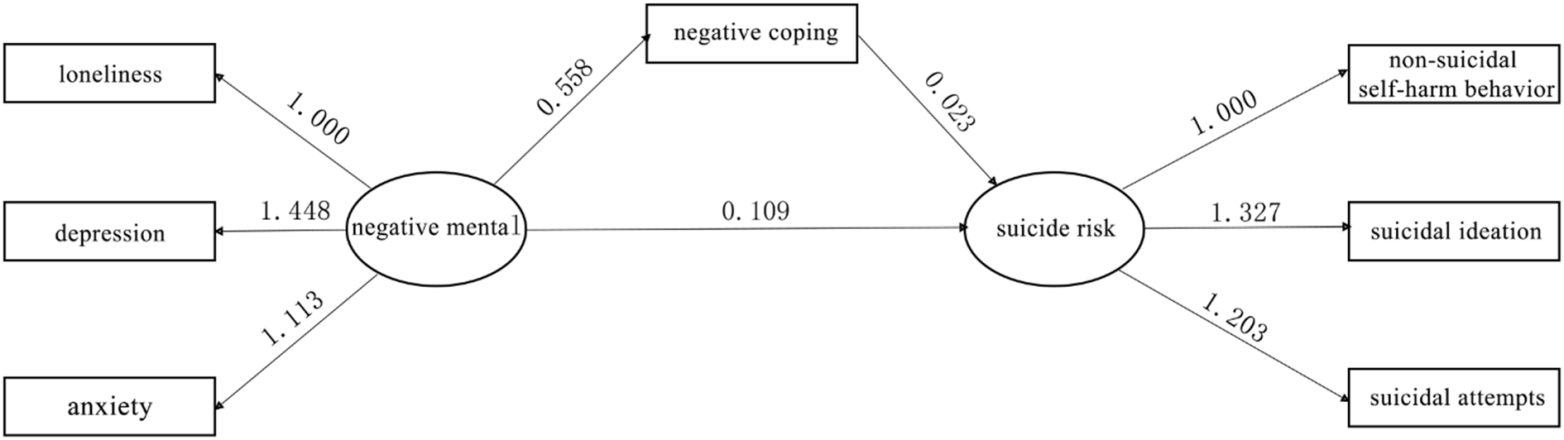
Path model of the relationships between negative mental, negative coping style and suicide risk.

Structural equation modeling was employed to examine direct and indirect effects of predictor variables using WLSMV method. Compared to the criteria of goodness-of-fit statistics, the model was characterized by a good fit: GFI = 0.999, AGFI = 0.997, CFI = 0.996, RMSEA = 0.029 and all paths were at a significant level of 0.05. Figure 1 shows the path analytical model with associated standardized regression weights.

Table 3 shows the path effects of the relationships between negative mental, negative coping style and suicide risk. Our findings revealed that negative coping was a mediating variable that was positively influenced by negative mental ($\beta =0.558, P<0.001$). Negative coping was also positively and directly associated with suicide risk ($\beta =0.023,P<0.001$). Meanwhile, negative mental was indirectly and positively associated with suicide risk ($\beta =0.013,P<0.01$) and directly and positively associated with suicide risk ($\beta =0.109,P<0.001$). Based on the fitting results of the mediation model, bias-corrected nonparametric percentile bootstrap method was used to test for the confidence interval. Here, the repeated sampling was performed 5000 times. Table 3 shows that all the path effects (direct and indirect) are significant as zero falls outside the confidence interval, indicating reliable path effects.

**Table 3:** Path effects of negative mental, negative coping style and suicide risk.

Path	Standardized coefficient	*95%CI*^a^	Bias-corrected *95%CI*^b^
Negative coping style ← Negative mental	0.558	[0.499, 0.676]	[0.486, 0.642]
Suicide risk ← Negative mental	0.109	[0.080, 0.138]	[0.071, 0.272]
Suicide risk ← Negative coping style	0.023	[0.007, 0.039]	[0.005, 0.036]
Suicide risk ← Negative coping style ← Negative mental	0.013	[0.005, 0.021]	[0.003, 0.021]

^a^95% confidence interval calculated for the percentile method; ^b^95% confidence interval calculated for bootsrap method.

## Discussion

China has experienced dramatic urbanization in recent decades. In the context of massive rural-to-urban migration, Shenzhen grew from a small village to a highly urbanized city. As a vital workforce in the urbanization process, however, migrant workers have received insufficient attention with regards to their mental health. Mental illness and suicidal behaviors of migrant workers posed serious challenges to China’s healthcare system. Their negative mental has also created obstacles to the urbanization process of China. Therefore, understanding relationships between related risk factors and suicide risk is of great significance for improving well-being of migrant workers and promoting urbanization in China more effectively. To our knowledge, this was the first study targeted specifically at the relationships between negative mental, negative coping styles and suicide risk among migrant workers.

In the large community sample covering 3095 migrant workers, we found that 2.81% participants reported some degree of suicide risk. Our results demonstrated that higher depression, anxiety, loneliness, and negative coping styles were associated with increased odds ratio of suicide risk. Consistent with previous studies, these findings indicated that negative mental (depression, loneliness, anxiety) and negative coping styles were independently risk factors of suicide among migrant workers^36–38^.

Structural equation modeling was constructed to further examine the relationships of negative mental, negative coping style and suicide risk. Consequently, we had three main findings. First, we found that higher negative mental (including depression, anxiety and loneliness) are strongly associated with higher suicide risk among migrant workers. Second, we found that negative coping styles are strongly associated with higher suicide risk. Third, we also found a positive correlation between negative mental and negative coping styles, indicating an indirect path effect between negative mental and suicide risk. These findings are discussed below in the context of emotional psychology.

Negative mental is directly related to suicide risk among migrant workers. This finding supported previous research that negative mental health was associated with a greater risk of suicide among migrant workers^10,14,15,39^. Although previous literature has pointed to the importance of stressful social contexts that place migrant workers at greater risk of suicide^40,41^, we suggested that negative mental should also be a central focus in understanding suicide risk in migrant workers. From the perspective of social psychology, negative mental can affect one’s social relationship with others. For example, individuals with depression and anxiety disorders were prone to suicidal ideation and behaviors due to their defects in emotional communication^42^. Previous findings also asserted that people from rural China who experienced loneliness lacked a sense of belongingness, which may result in a higher rate of suicide risk^43^. In addition, among common theories in the explanation between negative mental and suicide risk, rumination plays an essential role. Wang reported a significant association of negative mental with suicidal ideation among college students beyond the effects of rumination^44^. It is possible that migrant workers with negative mental were more vulnerable to the negative effects of perceived stress, and this kind of reflective rumination process eventually increased their suicide risk.

The association between negative coping styles and suicide risk also received attention in the present study. Our findings suggested that negative coping styles are positively associated with suicide-related behaviors, which is consistent with prior research^45^. In the current study, subjects who have exhibited suicidal behaviors adopted negative coping styles more frequently than the controls. Previous retrospective study revealed that people who use helpless and submissive coping styles had increased probability of suicide^46^. Using the Chinese rural youth suicide data, Li also found coping skills as a mediating factor between life stress and suicide ideation^17^. In the face of a variety of stressors that stemmed from poor living conditions, poor social welfare, social inequity and discrimination, migrant workers who engaged in negative coping styles may have a higher rate of suicide risk^39^.

Additionally, as expected a positive relationship between negative mental and negative coping style was revealed. Previous research on the general population has shown that depressed individuals were more likely to pursue negative coping behaviors^47^. Our findings confirmed that loneliness and anxiety were also associated with negative coping styles. Furthermore, the present study highlighted that the pathways or mechanisms involved in negative mental, negative coping styles and suicide risk appear to be complex, as negative coping styles can partially mediate the association between negative mental and suicide risk. Experiencing negative mental cannot be an independent risk factor for suicide among migrant workers, as some people may respond to the same negative mental in a different way. It means that negative mental also indirectly affected suicide risk via the mediator: negative coping styles. For example, symptoms of depression can cause apathy which eventually leads to inactivity, and prolonged inactivity is proved to be central in developing suicidal behaviors^48^. We suggested that this mediation model should be further explored in randomized clinical trials.

Our results showed that negative mental (depression, anxiety, loneliness) positively affected suicide risk directly and indirectly. Therefore, to lower the likelihood of future suicide among migrant workers, targeted interventions for improving mental health are needed. We suggest the following recommendations at both political and clinical level.

At the political level, structural efforts should be made to relax rural-to-urban migrant workers from *hukou* restrictions, which produced the majority of stressors among migrant workers. In recent years, China has begun to engage in the reform of the household registration system (*hukou)*, focusing on reducing the inequality of social welfare between migrant workers and urban residents. Fairer treatment should be given to ensure that urban public services and social security are no longer only related to household registration. And it’s important to provide migrant workers with medical insurance that covers mental health services. At the clinical level, basic preventative strategies consist of establishing more accessible social networks, more valuable psycho-educational programs, more complete mental health services and more accurate negative mental screening methods. Additionally, individual privacy should be carefully protected when migrant workers seek help from mental health services.

The present study also underlined the importance of promoting effective coping styles in suicide prevention. Regular in-service training promoting effective coping styles should be provided to migrant workers, such as physical activity, listening to music, seeking family support, spending time with friends, and consulting a trained mental health provider or counselor.

Conclusions based on the present results should be made cautiously in light of several important limitations. First, causal relationships between negative mental, negative coping, and suicide risk cannot be warranted with the cross-sectional data. Second, there are other mental health problems such as impulsion related to suicide risk that have not been included in this study. In addition, the study relied on self-report measures of mental health, which may involve the risk of various bias such as social desirability bias. Our later studies should consider more mental illnesses and utilize multiple methods (e.g., qualitative interview) to investigate the mental health of migrant workers. Third, the measure of suicide risk was binary (yes/no), rather than continuous. Future studies should conduct a continuous measure for suicide risk. Finally, we didn’t analyze whether stressors participated in the causal strain between negative mental, negative coping styles and suicide risk. According to the “stress-sensitization” model of suicide theory, the interaction of stress and negative mental could increase the suicide risk^49^. Future studies should conduct moderation analyses to understand the relationships between life stressors, negative mental, negative coping styles and suicide risk.

## Conclusions

The present study provided novel information about relationships between suicide risk, negative mental and negative coping style based on a large sample of migrant workers. The hypotheses in the study were verified in the results. Specifically, negative mental (depression, loneliness and anxiety) had a direct positive impact on suicide risk and indirectly affected suicide risk through negative coping style. Negative coping style also had a significant positive effect on suicide risk. Together, these key findings elucidated partial psychological mechanisms involved in suicide among migrant workers. To prevent suicidal behaviors among migrant workers, an improvement of mental health requires policy change, medical system support and enhanced clinical help. It’s also of great necessity to provide tailored assistance for cultivating adaptive coping styles among migrant workers. Future research would be feasible and valuable if many interventions are attempted for improving the mental health well-being of rural-to-urban migrant workers.
